# Meta-analysis and sustainability of feeding slow-release urea in dairy production

**DOI:** 10.1371/journal.pone.0246922

**Published:** 2021-02-12

**Authors:** Saheed A. Salami, Colm A. Moran, Helen E. Warren, Jules Taylor-Pickard

**Affiliations:** 1 Solutions Deployment Team, Alltech (UK) Ltd., Stamford, United Kingdom; 2 Regulatory Affairs Department, Alltech SARL, Vire, France; 3 Alltech Biotechnology Centre, Dunboyne, Ireland; University of Illinois, UNITED STATES

## Abstract

Slow-release urea (SRU) is a coated non-protein nitrogen (NPN) source for providing rumen degradable protein in ruminant nutrition. A meta-analysis was conducted to evaluate the effects of replacing vegetable protein sources with SRU (Optigen^®^, Alltech Inc., USA) on the production performance of dairy cows. Additionally, the impact of SRU supplementation on dairy sustainability was examined by quantifying the carbon footprint (CFP) of feed use for milk production and manure nitrogen (N) excretion of dairy cows. Data on diet composition and performance variables were extracted from 17 experiments with 44 dietary comparisons (control *vs*. SRU). A linear mixed model and linear regression were applied to statistically analyse the effect of SRU on feed intake and production performance. Feeding SRU decreased (*P* < 0.05) dry matter intake (DMI, -500 g/d) and N intake (NI, -20 g/d). There was no significant effect (*P* > 0.05) on milk yield, fat-corrected milk, energy-corrected milk, and milk fat and protein composition. However, SRU supplementation improved (*P* < 0.05) feed efficiency (+3%) and N use efficiency (NUE, +4%). Regression analyses revealed that increasing SRU inclusion level decreased DMI and NI whereas increasing dietary crude protein (CP) increased both parameters. However, milk yield and feed efficiency increased in response to increasing levels of SRU inclusion and dietary CP. The NUE had a positive relationship with SRU level whereas NUE decreased with increasing dietary CP. The inclusion of SRU in dairy diets reduced the CFP of feed use for milk production (-14.5%; 373.13 vs. 319.15 g CO_2_ equivalent/kg milk). Moreover, feeding SRU decreased manure N excretion by 2.7% to 3.1% (-12 to -13 g/cow/d) and N excretion intensity by 3.6% to 4.0% (-0.50 to -0.53 g N/kg milk). In conclusion, feeding SRU can contribute to sustainable dairy production through improvement in production efficiency and reduction in environmental impacts.

## Introduction

There is an increasing interest to optimize the utilization of dietary protein in dairy cows to enhance production efficiency, reduce feed cost and mitigate environmental impacts of dairy production. Feeding dietary protein to dairy cows involves formulating diets with a balance of rumen degradable protein (RDP) and undegradable protein (RUP) to meet the nutritional requirements of the animal. Ruminal hydrolysis of RDP releases ammonia (NH_3_) into the rumen, and when synchronized with fermentable energy is used to synthesize microbial crude protein (MCP) [1]. The MCP is a high-quality protein with high apparent digestibility and balanced amino acid (AA) profile [2]. The MCP contributes most of the CP flowing into the small intestine, and the combination of MCP and RUP constitutes the metabolizable protein that is digested and absorbed in the small intestine to meet the AA requirement of dairy cows [3].

Urea is an NPN compound that can be used to supply RDP in ruminant rations [4]. The economical cost of urea has increased interest in its utilization as a partial replacement of plant protein sources, such as soybean meal (SBM), to supply RDP [5]. However, the utilization of urea in ruminant nutrition is limited due to its rapid hydrolysis to NH_3_ in the rumen, exceeding the rate of carbohydrate fermentation in the rumen. The asynchrony between rumen NH_3_ production and available fermentable energy could exert a negative effect on the efficiency of MCP synthesis [1]. Consequently, this condition reduces the amount of MCP outflow which may impair the availability of metabolizable protein for milk production [3]. Moreover, rapid hydrolysis of urea in the rumen can reduce N utilisation efficiency (NUE, milk N as a percentage of total N intake) and increase N excretion [1]. The rapid hydrolysis of urea could also elevate blood NH_3_ concentration and increase the risk of NH_3_ toxicity [4]. To alleviate the problems associated with feeding feed-grade urea, coating technologies have been utilized to develop slow-release urea (SRU) products that could control urea degradation and release of NH_3_ into the rumen. This could improve the synchronisation of ruminal production of NH_3_ with energy digestion and reduce the metabolic cost of detoxifying NH_3_ to urea in the liver [5]. An extensive review of literature has demonstrated the efficacy of SRU as an NPN source that improves the efficiency of rumen N capture, microbial protein synthesis, fibre digestion and thus improved the production of ruminant milk and meat [5].

The environmental impact of dairy production systems is mainly associated with greenhouse gas emissions (GHG) and N excretion [6,7]. Carbon dioxide (CO_2_), methane (CH_4_), and nitrous oxide (N_2_O) are the main GHG emissions associated with agriculture and are conventionally expressed in terms of CO_2_ equivalent (CO_2_-eq) per unit of product. Life cycle assessments have shown that global emissions from milk production contribute 1.4 gigatonnes CO_2_-eq [8] and the global dairy sector accounts for approximately 4% of the total global anthropogenic GHG emissions [9]. Feed represents a major input of GHG in carbon footprint (CFP) accounting of dairy systems, and the related feed emissions are derived mainly from fertilization of feed crops, deposition of manure on pastures and land-use changes [8]. Formulating dairy diets with a lower CFP is a potential strategy to reduce the overall emission intensity of milk production [10]. Following this strategy, the use of SRU as a partial replacement for SBM and cottonseed meal reduced the feed CFP of sheep and dairy buffalo, respectively, [11,12]. Furthermore, ruminants have a relatively low NUE, excreting 60 to 90% of ingested N in manure—urine and faeces [13]. Manure N excretion on dairy farms has attracted increasing environmental concerns because of its effects on water quality through nitrate leaching and eutrophication, and subsequent release of gases such as NH_3_ and N_2_O, which negatively affect air quality and causes global warming, respectively [14,15]. The positive effect of SRU to improve N capture in the rumen could increase the amount of N retained for milk production and improve the NUE of dairy cows. Thus, it can be expected that feeding SRU can improve dairy sustainability by reducing the feed CFP and manure N excretion.

To our knowledge, there is no existing information on the objective review quantifying the effects of SRU in dairy production. A meta-analysis is a quantitative technique that can be used to systematically combine datasets from multiple studies with different experimental designs, heterogeneity and treatment effects, and allows for providing evidence-based conclusions from a body of research [16,17]. In a recent meta-analysis study, we have demonstrated that partial replacement of plant protein sources with dietary SRU improved production performance of growing and finishing beef cattle [18]. Thus, the objective of the present study was to apply a meta-analytic technique to evaluate the retrospective effects of SRU supplementation on the production performance of dairy cows. Additionally, the impacts of SRU supplementation on feed CFP and manure N excretion were examined as dairy sustainability metrics.

## Materials and methods

### Literature search strategy and study selection

**Fig 1:**
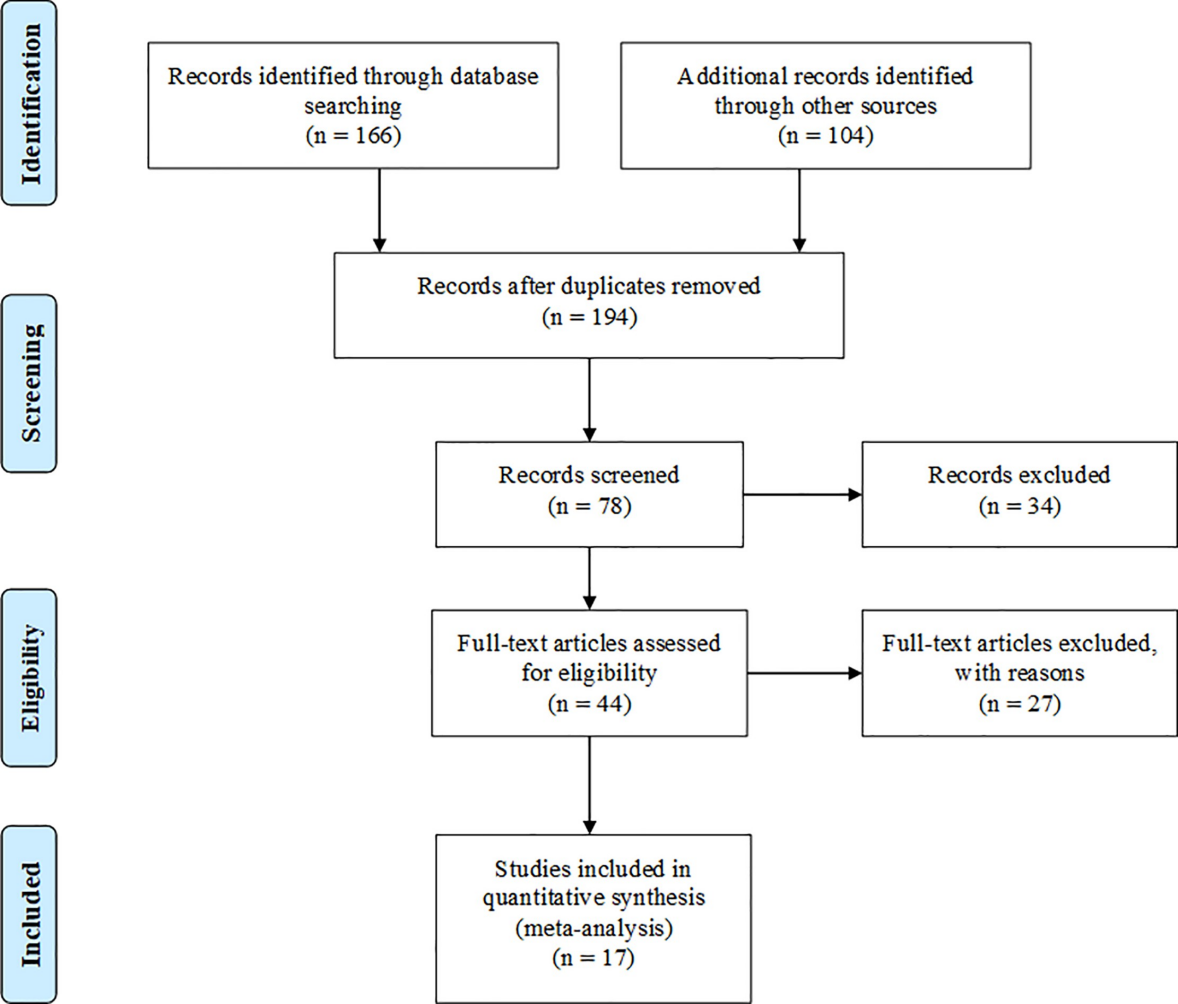
A PRISMA flow diagram detailing the literature search strategy and study selection for the meta-analysis.

This meta-analysis is reported following the Preferred Reporting Items for Systematic Reviews and Meta-Analyses (PRISMA) Statement [19] as presented in Fig 1. The meta-analysis was performed to evaluate the effect of a commercial SRU product (Optigen^®^, Alltech Inc., Nicholasville, KY, USA) on production performance of dairy cows. The SRU product consists of urea evenly coated with a semi-permeable vegetable fat matrix containing 88% urea (41% N, 256% CP) and 11–12% fat [18]. The fat coating in the SRU slows the dissolution of urea, reducing the rate of urea conversion to NH_3_ in the rumen [20]. A literature search was conducted using online academic databases (Google Scholar, Scopus, PubMed, CAB Direct, Web of Science, and Mendeley) to retrieve published studies evaluating the effect of the SRU product in dairy cows. The search strategy included the following words “dairy cow”, “slow-release urea”, “polymer-coated urea”, “Optigen”, and “milk production”.

No date restriction was applied to the literature search to encompass the entire duration that the SRU product has been used in dairy nutrition research. Additionally, the company’s bibliography database was searched to retrieve published and unpublished trial reports that evaluated the effect of SRU in dairy cows. The unpublished trial reports are linked to the company’s research team, which allows for retrieving more information if required. The studies were selected after screening for the following criteria: (1) the trial was reported in English; (2) the experiment was conducted in dairy cattle breeds; (3) studies contain at least one control diet without SRU supplement and a diet supplemented with the SRU product as a partial replacement for plant protein sources; (4) the SRU dosage was reported; (5) information on feed ingredient composition of diets was provided or available on request from authors; and (6) information on production performance parameters (dry matter intake (DMI), milk yield and composition) was reported or available on request from authors. Based on these criteria, 17 studies were selected for inclusion in the meta-analysis. The selected studies consist of 11 peer-reviewed publications and 6 unpublished studies presented at international conferences. Details of the experimental studies included in the meta-analysis are presented in Table 1.

**Table 1:** Description of experimental studies used in the meta-analysis examining the effect of SRU supplementation on the performance of dairy cows.

Reference	Country	Breed	Feeding regimen	No of animals (control)	No of animals (SRU)	SRU dose % DM	Duration (day)
Abdel-Raouf et al. [21]	Egypt	Holstein	TMR (Corn silage/corn-based diet)	4	4	0, 0.26, 0.53	84
Agovino [22]	Italy	Holstein-Friesian	TMR (Corn silage/grass hay-based diet)	155	155	0, 0.36	40
Akay et al. [23] (Trial 1)	USA	Holstein	TMR (Corn silage/corn-based diet)	103	103	0, 0.94	77
Akay et al. [23] (Trial 2)	USA	Holstein	TMR (Corn silage/corn-based diet)	120	120	0, 0.67	30
Gadegaonkar et al. [24]	India	Gir, Gir x Holstein, Gir x Jersey	Component feeding (Hay, green grass, concentrate mixture)	6	6	0, 1	180
Galo et al. [25]	USA	Holstein	TMR (Corn silage/corn-based diet)	8	8	0, 0.77	57
Giallongo et al. [26]	USA	Holstein	TMR (Corn silage-based diet)	12	12	0, 0.40	112
Inostroza et al. [27]	USA	Holstein	TMR (Corn silage/alfalfa silage-based diet)	2368	2368	0, 0.40	60
Miranda et al. [28]	Brazil	Holstein	TMR (Corn silage-based diet)	8	8	0, 0.75	112
Neal et al. [29]	USA	Holstein	TMR (Alfalfa hay/corn silage/corn-based diet)	12	12	0, 0.49	112
Santiago et al. [30]	Brazil	Holstein x Zebu	TMR (Sorghum silage/corn-based diet)	8	8	0, 1.12	60
Santos et al. [31]	Brazil	Holstein	TMR (Corn silage/citrus pulp-based diet)	18	18	0, 0.61	63
Sinclair et al. [32]	United Kingdom	Holstein-Friesian	TMR (Grass silage/corn silage/triticale-based diet)	42	42	0, 0.55	105
Souza et al. [33]	Brazil	Holstein	TMR (Corn silage/grass haylage-based diet)	17	17	0, 0.40	30, 60
Stewart et al. [34]	USA	Holstein	TMR (Corn silage-based diet)	6	6	0, 0.64	42
Tye et al. [35]	USA	Holstein	TMR (Corn silage/alfalfa hay/corn-based diet)	8	8	0, 0.45, 0.46	84
Varga and Ishler [36]	USA	Holstein	TMR (Corn silage-based diet)	60	60	0, 0.44	90

SRU: Slow-release urea; TMR: Total mixed ration.

### Data extraction

Data were extracted from the selected studies into a spreadsheet database. The database consisted of 44 control and SRU dietary comparisons. Data were extracted for variables on diet composition, feed intake and production performance response. The diet composition variables included the inclusion levels of feed ingredients and SRU, and diet CP level (%). The feed intake and production performance variables included DMI (kg/d), N intake (NI, g/d), milk yield (kg/d), fat-corrected milk (FCM, kg/d), energy-corrected milk (ECM, kg/d), milk component (% fat and % protein), milk component yield (protein and fat yield, g/d), feed efficiency (kg FCM/kg DMI) and NUE (%). All data extracted from the studies into the database were standardized to the same units of measurement of the respective variable. If not reported, feed intake or production performance variables were calculated using the following equations:

NI=[DMI×(%CP/6.25)] [1]

%milkcomponent=(milkcomponentyield/milkyield)×100 [2]

Milkcomponentyield=(%milkcomponent×milkyield)/100 [3]

FCM=(0.35×milkyield)+(18.57×milkfatyield) [4]

ECM=(0.327×milkyield)+(12.95×milkfatyield)+(7.65×milkproteinyield) [5]

FE=FCM/DMI [6]

NUE(%)=[(milkproteinyield/6.25)/NI]×100 [7]

where FCM is milk adjusted to 3.5% fat and ECM is milk adjusted to 3.5% fat and 3.0% true protein. The DMI, milk yield, FCM and ECM were expressed as kg/d while NI was expressed as g/d.

### Feed carbon footprint and manure nitrogen excretion

The feed database was created from common feed raw materials used in the control and SRU diets in all the selected studies. The feed database was populated with the CFP values (including land-use changes) of feed raw materials retrieved from the Dutch FeedPrint software developed by Wageningen University and Research, The Netherlands [37]. The FeedPrint was developed to gain insight into the GHG emissions during the production and utilization chain of feed and to identify mitigation options [37]. In exceptional cases when CFP of the feed raw material was not included in the FeedPrint, the CFP was retrieved from the Plurimix^®^ diet formulation software (Fabermatica, Ostiano, Italy). The CFP of common feed raw materials included in the feed database are presented in S2 Table. The contribution of the feed raw materials to the feed CFP was estimated by multiplying the inclusion level of the raw material and the CFP per of kg raw material (g CO_2_-eq/kg). The average feed CFP was calculated and expressed as g CO_2_-eq/kg diet. The CFP of feed use for milk production was calculated by dividing the feed CFP by the feed efficiency and the result was expressed in g CO_2_-eq/kg milk. Additionally, the feed database was used to estimate the relative changes in the inclusion level of common feed raw materials between the control and SRU diets.

Documented static equations were used to calculate manure N excretion (Nex) and N excretion intensity (Nexi) based on the results of feed intake and production performance variables (DMI, NI, and milk yield) obtained from the meta-analysis of control and SRU diets. The Nex expressed the absolute amount of N excreted per day (g/cow/d) while the Nexi expressed the amount of N excreted per unit of milk yield (g N/kg milk). The following referenced equations were used to calculate Nex Kebreab et al. [38], Tomlinson et al. [39], Weiss et al. [40]:

Nex=10+(0.28×NI)+20+(0.38×NI); [8]

Nex=0.778×NI−(6.93×DMI)+122.6; [9]

Nex=51+(NI×0.64)−(0.94×milkyield); [10]

### Statistical analysis

The effect of SRU supplementation on feed intake and production performance parameters were subjected to statistical analysis using a linear mixed model. The treatment effect was included as a fixed effect, experimental duration as covariates and the study effect was included as a random effect [17]. The number of animals was used as a weighing factor for the analysis [41,42]. Results of treatment effect are reported as least square means for the control and SRU diets. Significance of treatment effect was declared when *P* < 0.05.

Furthermore, regression analyses were performed to investigate the relationship between feed intake and production performance in response to SRU inclusion level and dietary CP content. Each of the feed intake and production performance parameters was considered as the respective dependent variable while the SRU inclusion level and dietary CP content were considered as predictive/independent variables. The number of animals was used as a weighing factor for the regression analyses. The model accuracy was evaluated by estimating the residual error as root mean square error (RMSE) and adjusted R^2^_._ The intercept and slope coefficients, and their respective standard error and significant levels are reported.

The linear mixed model and regression analyses were performed using the SPSS software (IBM Statistics version 22). The presence of publication bias in the studies used for the meta-analysis was examined both graphically with funnel plots and statistically with Begg’s test [43] using the Comprehensive Meta-analysis software (version 3, Biostat Inc., USA). Publication bias assessed with the Begg’s test was considered significant when *P <* 0.05.

## Results

### Diet composition and study characteristics

As shown in Table 1, total mixed rations (TMR) were the dominant feeding regimen in 16 of the 17 studies included in this meta-analysis. Reformulating dairy diets with SRU concentrates the N fraction of the diet, which creates dry matter space for more fibre and energy sources to be included. Following this diet reformulation strategy, the feed database developed in this meta-analysis indicated that the average inclusion levels of plant protein sources were relatively lower in the SRU diets (S3 Table) whereas the inclusion levels of energy and fibre sources increased in the SRU diets (S4 Table). Compared to the control diets, the SRU diets contained lower inclusion of plant protein sources including SBM (-20.8%), canola/rapeseed meal (-29.2%), alfalfa haylage (-32.7%), cottonseed cake (-62.0%) and corn distillers’ grains (-18.7%) (S3 Table). This was accompanied by an increase in fibre and energy sources in the SRU diets, mainly corn products (+9.5%), grass haylage (+5.6%), citrus pulp (+26.1%) and wheat bran (+119.9%).

The summary statistics of diet, feed intake and production performance variables included in the meta-analysis are presented in Table 2. The average inclusion level of SRU across all SRU diets was 0.58% DM diet. The CP content of diets varied between 11.8% and 23.7%. Similarly, the DMI and NI varied across diets, with an average value of 22.9 kg/d and 633.8 g/d, respectively. Milk yield, FCM and ECM averaged 32.16, 32.98, and 33.30 kg/d, respectively. The averages observed for milk components were milk fat (3.59%), milk fat yield (1.19 kg/d), milk protein (3.12%) and milk protein yield (1.03 kg/d). There were large differences in feed efficiency (0.58 to 1.89) and NUE (17.2 to 34.7%) of the cows used in studies included in the meta-analysis.

**Table 2:** Descriptive statistics of diet, feed intake and production performance variables extracted from studies included in the meta-analysis.

Item	n	Mean	Minimum	Maximum	SD
*Dietary variables*					
SRU inclusion level (% DM diet)	22	0.58	0.26	1.12	0.23
Diet CP content (% DM)	44	17.16	11.82	23.74	2.67
*Feed intake*					
Dry matter intake (DMI, kg/d)	44	22.92	10.33	29.90	4.12
Nitrogen intake (NI, g/d)	44	633.84	195.36	882.84	151.50
*Performance*					
Milk yield (kg/d)	44	33.46	5.80	43.80	9.11
Fat-corrected milk (FCM, kg/d)	44	33.80	5.99	44.43	8.84
Energy-corrected milk (ECM, kg/d)	44	34.24	6.27	45.10	8.93
Milk fat (%)	44	3.59	2.71	4.51	0.37
Milk fat yield (kg/d)	44	1.19	0.21	1.59	0.31
Milk protein (%)	44	3.12	2.79	3.86	0.21
Milk protein yield (kg/d)	44	1.03	0.21	1.40	0.27
^3^Feed efficiency	44	1.45	0.58	1.89	0.28
Nitrogen use efficiency (%)	44	26.00	17.24	34.66	4.67

SRU: Slow-release urea; CP: Crude protein; SD: Standard deviation.

### Feed intake and production performance

The effect of feeding SRU on feed intake and production performance of dairy cows are presented in Table 3. The SRU diets decreased (*P* < 0.05) DMI (-500 g/d) and NI (-20 g/d). However, SRU supplementation did not influence (*P* > 0.05) milk yield, FCM, ECM, milk fat and protein percentages or milk protein and fat yields. The partial replacement of plant protein sources with SRU significantly improved the feed efficiency (+3%) and NUE (+4%) of dairy cows. The symmetrical shape of the funnel plots and results of the Begg’s test indicated that there was no significant publication bias in the studies used for meta-analysis evaluation of feed intake and production performance variables (S1–S3 Figs).

**Table 3:** Effect of feeding slow-release urea on feed intake and production performance of dairy cows.

Item	Diet		SEM	*P*-value
	CON	SRU		
*Feed intake*				
Dry matter intake (DMI, kg/d)	22.97	22.47	0.335	0.004
Nitrogen intake (NI, g/d)	625.57	605.67	12.865	0.009
*Production performance*				
Milk yield (kg/d)	32.16	32.46	0.437	0.307
Fat-corrected milk (FCM, kg/d)	32.98	33.16	0.412	0.381
Energy-corrected milk (ECM, kg/d)	33.30	33.52	0.402	0.284
Milk fat (%)	3.67	3.66	0.023	0.681
Milk fat yield (g/d)	1169.85	1173.84	14.653	0.655
Milk protein (%)	3.14	3.15	0.025	0.931
Milk protein yield (g/d)	998.01	1007.19	11.927	0.331
Feed efficiency	1.41	1.45	0.022	0.013
Nitrogen use efficiency (%)	25.28	26.41	0.534	0.016

CON: Control treatment; SRU: Slow-release urea treatment.

Table 4 shows the relationship between feed intake and production performance in response to inclusion level of SRU and dietary CP content. Regression analyses revealed that increasing SRU inclusion level reduced DMI (*P* < 0.001, R^2^ = 0.536) and NI (*P* < 0.001, R^2^ = 0.761) whereas increasing dietary CP content increased both parameters. The model showed a low correlation for predicting a relationship of FCM, ECM, milk fat and protein yield with the predictor variables (SRU level and Diet CP content). However, increasing SRU inclusion level and diet CP content had a positive relationship to increase milk yield (*P* < 0.001, R^2^ = 0.307) and feed efficiency (*P* < 0.001, R^2^ = 0.427). Moreover, the NUE (*P* < 0.001, R^2^ = 0.542) increased with increasing SRU level while diet CP content had a negative relationship on NUE.

**Table 4:** Linear regression of the relationship of feed intake and production performance in response to the inclusion level of slow-release urea (SRU, %DM diet) and dietary crude protein content (CP, %DM).

Response parameter	^1^Parameter estimates									^2^Model estimates			
	Intercept			SRU level			Diet CP level						
	a	SE	*P-*value	b	SE	*P-*value	b	SE	*P-*value	RMSE	Adj-R^2^	*P*-value
*Feed intake*													
Dry matter intake (DMI, kg/d)	21.23	0.445	<0.001	-12.33	0.235	<0.001	0.64	0.024	<0.001	1.604	0.536	<0.001
Nitrogen intake (g/d)	-49.22	12.334	<0.001	-343.92	6.516	<0.001	54.83	0.670	<0.001	44.440	0.761	<0.001
*Production performance*													
Milk yield (kg/d)	10.44	0.704	<0.001	4.52	0.372	<0.001	1.32	0.038	<0.001	2.535	0.307	<0.001
FCM (kg/d)	21.58	0.729	<0.001	3.67	0.385	<0.001	0.79	0.040	<0.001	2.626	0.138	<0.001
ECM, kg/d)	23.94	0.716	<0.001	3.50	0.378	<0.001	0.659	0.039	<0.001	2.579	0.109	<0.001
Milk fat (%)	5.510	0.037	<0.001	-0.14	0.019	<0.001	-0.10	0.002	<0.001	0.133	0.447	<0.001
Milk fat yield (g/d)	965.07	27.19	<0.001	112.43	14.364	<0.001	17.59	1.476	<0.001	97.970	0.062	<0.001
Milk protein (%)	5.469	0.034	<0.001	-0.14	0.018	<0.001	-0.13	0.002	<0.001	0.121	0.643	<0.001
Milk protein yield (g/d)	1050.35	22.276	<0.001	74.02	11.768	<0.001	0.05	1.209	0.965	80.26	0.012	<0.001
Feed efficiency	0.959	0.033	<0.001	0.83	0.017	<0.001	0.003	0.002	0.062	0.119	0.427	<0.001
Nitrogen use efficiency (%)	54.70	0.746	<0.001	11.70	0.394	<0.001	-2.082	0.041	<0.001	2.688	0.542	<0.001

^**1**^**Parameter estimates: a: Intercept constant; b: Slope coefficient; SE: Standard error.**

^2^Model estimates: RMSE: Root mean square error; Adj-R^2^: Adjusted R^2^.

### Dairy sustainability

**Fig 2:**
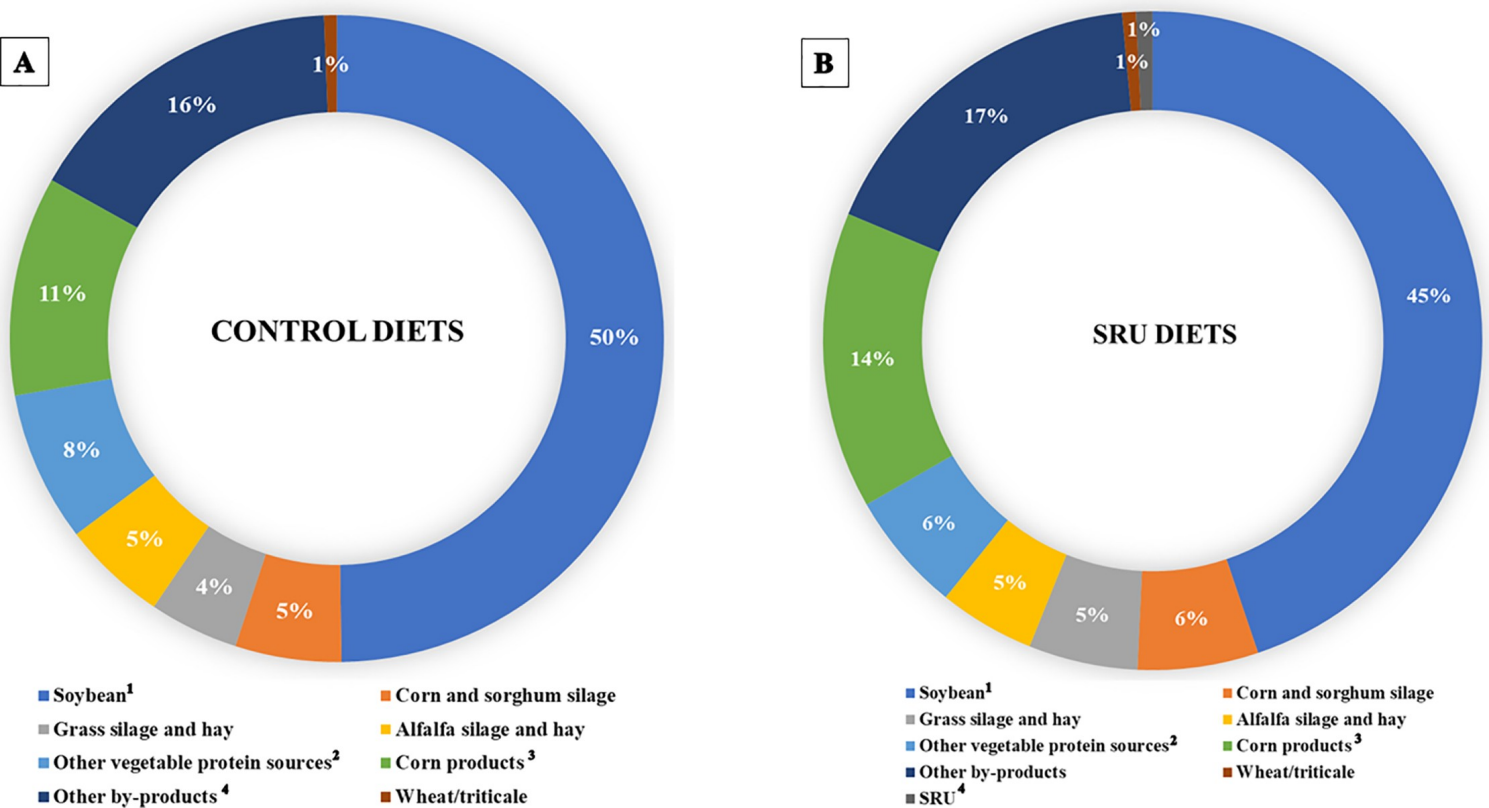
Contribution of common feed raw materials to the average carbon footprint of (A) control diets (B) Slow-release urea (SRU) diets. ^1^Soybean include soybean meal, toasted soybean, heated soybean seeds and soybean bypass. ^2^Other vegetable protein sources include canola meal, rapeseed meal, corn and wheat distillers’ grains, linseed meal, cottonseed cake and corn gluten meal. ^3^Corn products include ground corn, high-moisture corn, steam-flaked corn and cornmeal. ^4^Other by-products include sugar beet pulp, wheat straw, citrus pulp, wheat bran, rice bran, brewers’ grains, wheat middlings, cottonseed hulls, soybean hulls.

**Fig 3:**
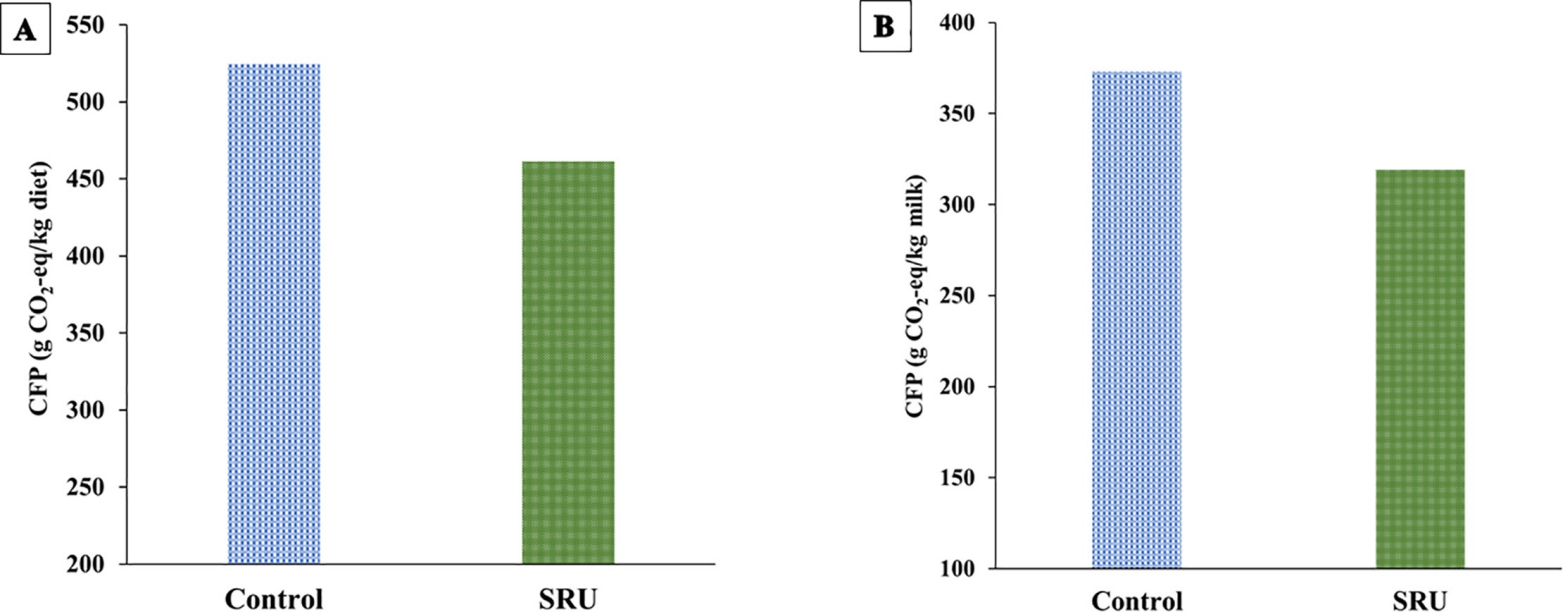
Effect of reformulating dairy diets with slow-release urea (SRU) on the (A) average carbon footprint (CFP) of dairy diets (B) average CFP of feed use for milk production.

The impact of feeding SRU on dairy sustainability was examined by estimating the CFP of feeds (Figs 2 and 3) and manure N excretion (Table 5). The results revealed that soybean products were the dominant contributor to the CFP of the dairy diets, accounting for 50% and 45% of the total CFP of the control and SRU diets, respectively (Fig 2). Corn products and by-products accounted for more than 10% of the feed CFP in both the control and SRU diets. Notably, the inclusion of SRU in the SRU diets contributed only 1% of the feed CFP (Fig 2). The partial replacement of plant protein sources with SRU decreased the CFP of the SRU diets (-12%; 461.50 *vs*. 524.62 g CO_2_-eq/kg diet) compared to the control diets (Fig 3A). Similarly, the CFP of feed use for milk production was lower for the SRU diets than the control diets (-14.5%; 319.15 *vs*. 373.13 g CO_2_-eq/kg milk) (Fig 3B).

Table 5 depicts the effect of feeding SRU on the manure N excretion (Nex) and N excretion intensity (Nexi). Equation estimates showed that feeding SRU decreased Nex by 2.7% to 3.1% (-12 to -13 g/cow/d). Similarly, equation estimates indicated that SRU supplementation reduced the amount of N excreted per unit of milk (Nexi) by 3.6% to 4.0% (-0.50 to -0.53 g N/kg milk).

**Table 5:** Calculated manure nitrogen excretion (Nex) and nitrogen excretion intensity (Nexi) based on the results of feed intake and production performance variables (DMI, kg/cow; NI, g/d; and MY, kg/d) obtained from the meta-analysis.

Parameters	Diet		Difference	% change
	CON	SRU		
*Nitrogen excretion (Nex*, *g/cow/d)*				
^1^NI	442.88	429.74	-13.13	-2.97
^2^NI + DMI	450.11	438.09	-12.02	-2.67
^3^NI + MY	421.13	408.12	-13.02	-3.09
^*4*^*Nitrogen excretion intensity (Nexi*, *g N/kg milk)*				
NI	13.77	13.24	-0.53	-3.86
NI + DMI	14.00	13.50	-0.50	-3.57
NI + MY	13.09	12.57	-0.52	-3.99

DMI: Dry matter intake; NI: Nitrogen intake; MY: Milk yield; CON: Control treatment; SRU: Slow-release urea treatment.

^1^Nex = 10 + (0.28 * NI) + 20 + (0.38 * NI); Kebreab et al. [38].

^2^Nex = 0.778 * NI − (6.93 * DMI) + 122.6; Tomlinson et al. [39].

^3^Nex = 51 + (NI * 0.64) − (0.94 * MY); Weiss et al. [40].

^4^Nexi = Nex/MY.

## Discussion

It has long been recognized that maximizing MCP synthesis is important, and that a deficiency of RDP could compromise the rumen NH_3_ level required to optimize MCP synthesis, resulting in a decrease in fibre digestibility, DMI and milk production [44]. The proprietary SRU product (Optigen^®^) evaluated in this study allows for reformulating dairy diets with less plant protein RDP sources. Our hypothesis is that this provides for a more sustained availability of NH_3_ in the rumen. This is expected to optimize the synchronization of ruminal production of NH_3_ with ruminal energy digestion to improve MCP synthesis. In an *in situ* trial conducted in cannulated animals, Akay et al. [23] demonstrated that N disappearance of SRU was more similar to that of SBM and slower than that of feed-grade urea. The authors further utilized a continuous culture fermenter simulating the rumen of Holstein dairy cows to demonstrate that a diet containing SRU at 0.66% DM increased bacterial protein synthesis and nutrient disappearance, suggesting improved nutrient digestion. These data indicated that SRU could be a viable controlled-release source of NPN in ruminant nutrition.

Over the last two decades, extensive research has been conducted on the application of SRU (Optigen^®^) in the ration of ruminants including dairy cows. The SRU has a N content of 41%, which supplies an equivalent CP content of 256%. This concentrates the N fraction of the diet and allows for reformulating diets with a lower inclusion level of plant protein sources such as SBM (CP, 40–48%) [18]. Reformulation of protein sources could influence feed cost, NUE and feed CFP [7,10]. In practice, formulating diets with SRU concentrates the N fraction of the diet and creates dry matter space for more fibre and energy sources to be included. Thus, reformulating diets with SRU can result in significant changes in the composition of raw materials in dairy diets.

In this context, a feed database was developed from the diet composition used in all the studies included in this meta-analysis. Several changes in feedstuff compositions were observed between the control and SRU diets, which reflects the fact that diet reformulation strategy depends on the variation in the local availability of feed raw materials. The present results showed that soybean products and canola/rapeseed meal, alfalfa, cottonseed cake and corn distillers’ grains were the main vegetable protein sources partially replaced with SRU. Notably, SBM was the most used RDP source and strategies that could reduce its inclusion level is attractive because of its volatile market price and potentially high CFP associated with land-use changes. Reduced use of SBM and the other aforementioned feedstuffs allowed for higher inclusion amounts of corn products, by-product feeds (sugar beet pulp, citrus pulp and wheat bran) and grass haylage. Corn products are the main energy source in most of the reformulated SRU diets, and this can be attributed to the aim of providing more fermentable energy that could enhance synchronization with NH_3_ to optimize MCP yield and greater milk production. Indeed, the changes observed in the composition of the experimental diets were consistent with the diet reformulation strategy applied in practice.

A multitude of studies have utilised meta-analysis to provide quantitative and research-based evidence on the efficacy of nutritional products or interventions in dairy cows [45–47]. To our knowledge, this is the first meta-analysis study that examined the effect of SRU supplementation on the production performance of dairy cows. Feeding SRU diets decreased DMI which explains most of the reduction in NI. The decrease in NI was also caused by a slightly lower CP content of the SRU diets compared to the control diets (16.60 *vs*. 16.75). Regression analyses indicated that increasing SRU inclusion level reduced NI. It is well documented that a decline in DMI may reduce nutrient intake and decrease milk production in dairy cows [48,49]. In contrast to this expectation, feeding SRU decreased DMI without a negative influence on milk yield and milk composition. A plausible explanation for this observation is that SRU provided for a more sustained release of NH_3,_ allowing for better synchronization with fermentable energy and enhanced rumen bacterial growth and microbial digestion. This effect could result in a greater supply of nutrients being absorbed for milk production.

Furthermore, feed efficiency is a significant measurement in dairy cows considering its link to profitability and environmental impacts of dairy production [50]. The present results revealed that feeding SRU diets improved the feed efficiency of dairy cows by 3%. This improvement in feed efficiency was driven by reduced DMI and a slight increase in milk yield. Recent knowledge has established that nutrition and breeding strategies could play critical roles in producing more efficient animals that require less feed for the same level of milk production [51,52]. This is consistent with the current indication that SRU improved the feed efficiency of dairy cows by reducing DMI without compromising milk yield. In this regard, discrepancies in feed efficiency can be attributed to physiological and biochemical mechanisms associated with digestion, energy capture and energy utilisation [52]. Moreover, the effect of SRU in enhancing N capture in the rumen and the better feed efficiency may be related to modulation in the rumen microbiome elicited by urea supplementation [53,54]. Li et al. [54] found that ruminal abundance of *Howardella* spp. and *Desulfobulbus spp*. increased in response to an increase in dietary N associated with feeding urea, suggesting that urea supplementation could affect ureolytic and sulfur-reducing abilities of the rumen bacteria community. Waghorn and Dewhurst [55] concluded that differences in feed efficiency can be associated with variation in rumen function, including duration and extent of rumen digestion, which influences the fermentation products (volatile fatty acids and MCP) that provide nutrients to the animal. In agreement with these assertions, dietary SRU has been shown to potentially improve MCP synthesis and ruminal digestion [23], which could explain the greater feed efficiency observed in cows fed the SRU diets. This is consistent with the positive relationship between feed efficiency and SRU inclusion level as indicated by the regression analyses.

The NUE is estimated as milk N yield as a percentage of total NI. Indeed, milk N yield is a function of milk yield and milk N content. Strategies that improve NUE are crucial for reducing N excretion and the environmental impacts of ruminant production [56–58]. Like other ruminant species, dairy cows have a relatively low NUE, reported at an average of 25% with an extensive variation (21 to 42%) between experiments [59]. In this study, the NUE varied between 17.2 and 34.7% with an average value of 26.0%, close to those reported by Whelan et al. [59]. The present results showed that SRU significantly improved NUE by 4%. The improvement in NUE observed in this study was mainly driven by a decrease in dietary NI, without negatively affecting milk yield or milk protein content. In agreement with our observation, Huhtanen and Hristov [47] reported that reducing NI is the most significant factor for increasing NUE, with a considerably larger effect on NUE compared to strategies that increase milk yield. The effect of SRU in improving NUE is expected to reduce N excretion in dairy cows [47]. The positive effect of SRU in improving N capture in the rumen could account for the better utilization of N for milk production, resulting in greater NUE. Moreover, the regression analyses confirmed that feeding SRU had a positive relationship with milk yield and NUE.

The environmental impacts of the dairy sector have attracted increasing concerns due to growing demand for dairy foods, accompanied by increasing GHG and N emissions. Major inputs associated with GHG emissions are primary energy (e.g. electricity and fuel) and feed (cultivation, processing and transport). Major GHG outputs are methane from enteric fermentation and manure management, and N_2_O from the reduction of manure nitrate in the soil. The present study focused on estimating feed CFP and manure N excretion as measures of dairy sustainability metrics. Feed emissions account for approximately 36% of GHG emissions from milk production [8]. In the current study, soybean products were the largest contributor to the CFP of dairy diets, accounting for almost half of the feed CFP. This suggests that the environmental impacts of dairy diets can be reduced considerably by replacing soybean products with low-carbon alternative protein feedstuff or nutritional solutions such as SRU. Feeding SRU diets in this study reduced feed CFP by 12% and the CFP of feed use for milk production by 14.5%. Interestingly, the CFP of feed use for milk production (319–373 CO_2_-eq/kg milk) found in this study was similar to the range of CFP values (180–340 CO_2_-eq/kg milk) reported for diets formulated for conventional dairy systems in Northern Europe and America [10]. This outcome is interesting considering that the feed CFP in this study was derived mostly from experiments conducted in America. Wilkinson and Garnsworthy [10] demonstrated that there is an opportunity to formulate diets with low CFP to reduce the CFP of feed use for milk production by up to 40% (from 239 to 142 CO_2_-eq/kg milk). This can be achieved by formulating the diet with grass silage and a high proportion of co-product feedstuffs with low CFP [10,60].

In the current study, the reduction in CFP was largely attributed to the partial replacement of soybean products with SRU. It is noteworthy that soybean products contributed 50% of the CFP of control diets while accounting for 45% of the CFP in SRU diets. In agreement with our results, the use of SRU as a partial replacement for SBM and cottonseed meal reduced the CFP of diets fed to sheep [12] and dairy buffalo [11]. Based on the reduction in feed CFP observed in this study, it can be estimated that the use of SRU in the diet of 1000 cows (milk yield = 40 kg/cow/d; annual milk production = 12,200 tonnes/year) could reduce the annual feed CFP for milk production by 646.81 tonnes CO_2_-eq/year. In perspective, this carbon saving is equivalent to taking 424 cars off the road in the UK, the average electricity use in 436 houses in the UK, or 1348 one-way transatlantic flights (per passenger) from London to New York.

Animal diets can exert a significant influence on enteric methane production in ruminants [61]. Methane is a potent GHG that accounts for a significant share of the overall CFP of milk production [9]. Thus, it is important to consider that feeding SRU to reduce feed CFP is not counterbalanced by an increase in enteric methane emission, which may offset any potential benefit on the environmental impacts of milk production. Notably, there is limited published information on the effect of feeding urea on enteric methane production. Alipour et al. [62] and Rebelo et al. [63] showed that feeding another form of SRU (urea coated with a blend of vegetable oils and polymers) and feed-grade (uncoated) urea did not affect enteric methane yield measured in an *in vitro* ruminal fermentation system or beef cattle, respectively. Moreover, the use of a life cycle assessment approach indicated that partial replacement of dietary SBM and cottonseed meal with SRU (Optigen^®^) did not affect enteric methane emission from sheep and dairy buffalo [11,12]. Although existing information suggests that dietary SRU may have little or no effect on enteric methane emission, there is a crucial need for future studies to evaluate the impact of feeding SRU on enteric methane under different dietary regimens to prevent environmental trade-offs in ruminant production.

Furthermore, minimizing manure N losses is a critical goal for sustainable N management in dairy production systems. Strategies that enhance NUE have been identified as a crucial approach to reduce Nex and N emission problems on dairy farms [58]. This could explain why the positive effect of SRU supplementation on NUE improvement is consistent with the reduction in the manure N excretion estimated from the three static equations. Based on the observed reduction in Nex, it can be estimated that feeding SRU to 1000 dairy cows could reduce annual N excretion by 4387.3 to 4792.5 kg N/year. The reduction in N excretion is expected to decrease NH_3_ volatilization, N_2_O losses and nitrate leaching from manure in animal barns, manure storage facilities and crop fields [6]. This is in agreement with the reduction in manure N_2_O emission reported when cottonseed meal was partially replaced with SRU in dairy buffalo [11].

## Conclusions

The meta-analysis showed that an average inclusion of SRU at 0.58% DM diet partially replaced vegetable protein sources in dairy diets, resulting in improved feed efficiency and NUE. Moreover, SRU supplementation improved the sustainability metrics of dairy production by reducing the CFP of feed use for milk production (-54 CO_2_-eq/kg milk) and manure N excretion (-12 to -13 g/cow/d). These results indicate that dairy diets can be reformulated with SRU to replace vegetable protein sources such as SBM while increasing energy sources such as corn. The increase in milk production efficiency observed in this study reduced the environmental impacts of milk production. Further work on whole-farm modelling is required to investigate the impacts of using SRU in different feeding scenarios on the cradle to farm-gate sustainability of milk production.

## Supporting information

S1 Fig Funnel plots of raw mean differences (difference in means) against their inverse standard errors and the associated significance (*P*-value for Begg’s test) for testing the publication bias of studies included in the meta-analysis for evaluating dry matter intake nitrogen intake, milk yield and fat-corrected milk. Open circles represent individual study comparisons included in the meta-analysis.
(TIF)

S2 Fig Funnel plots of raw mean differences (difference in means) against their inverse standard errors and the associated significance (*P*-value for Begg’s test) for testing the publication bias of studies included in the meta-analysis for evaluating energy-corrected milk, milk fat, milk fat yield and milk protein. Open circles represent individual study comparisons included in the meta-analysis.
(TIF)

S3 Fig Funnel plots of raw mean differences (difference in means) against their inverse standard errors and the associated significance (*P*-value for Begg’s test) for testing the publication bias of studies included in the meta-analysis for evaluating milk protein yield, feed efficiency and nitrogen use efficiency. Open circles represent individual study comparisons included in the meta-analysis.
(TIF)

S1 Table PRISMA checklist. (DOC)

S2 Table Carbon footprint (including land-use changes) of common feed raw materials used in studies included in the meta-analysis. (DOCX)

S3 Table Effect of diet reformulation with slow-release urea (SRU) on the average dietary inclusion levels of vegetable protein sources in studies used in the meta-analysis. (DOCX)

S4 Table Effect of diet reformulation with slow-release urea (SRU) on the average dietary inclusion levels of vegetable energy and fibre sources in studies used in the meta-analysis. (DOCX)

## References

[1] Calsamiglia S, Ferret A, Reynolds C, Kristensen NB, Van Vuuren A. Strategies for optimizing nitrogen use by ruminants. Animal. 2010;4(7):1184–96. 10.1017/S1751731110000911 22444616

[2] Schwab CG, Broderick GA. A 100-Year Review: Protein and amino acid nutrition in dairy cows. Journal of Dairy Science. 2017;100(12):10094–112. 10.3168/jds.2017-13320 29153157

[3] Owens F, Qi S, Sapienza D. Invited Review: Applied protein nutrition of ruminants—Current status and future directions. The Professional Animal Scientist. 2014;30(2):150–79.

[4] Kertz A. Urea feeding to dairy cattle: A historical perspective and review. The Professional Animal Scientist. 2010;26(3):257–72.

[5] Cherdthong A, Wanapat M. Development of urea products as rumen slow-release feed for ruminant production: a review. Australian Journal of Basic and Applied Sciences. 2010;4(8):2232–41.

[6] Wattiaux M, Uddin M, Letelier P, Jackson R, Larson R. Invited Review: Emission and mitigation of greenhouse gases from dairy farms: The cow, the manure, and the field. Applied Animal Science. 2019;35(2):238–54.

[7] Wilkinson J, Garnsworthy P. Impact of diet and fertility on greenhouse gas emissions and nitrogen efficiency of milk production. Livestock. 2017;22(3):140–4.

[8] Gerber PJ, Steinfeld H, Henderson B, Mottet A, Opio C, Dijkman J, Falcucci A, et al Tackling climate change through livestock: a global assessment of emissions and mitigation opportunities: Food and Agriculture Organization of the United Nations (FAO), Rome, Italy; 2013.

[9] Gerber P, Vellinga T, Opio C, Henderson B, Steinfeld H. Greenhouse gas emissions from the dairy sector. A Life Cycle Assessment Food and Agriculture Organization of the United Nations (FAO), Rome, Italy 2010.

[10] Wilkinson J, Garnsworthy P. Dietary options to reduce the environmental impact of milk production. The Journal of Agricultural Science. 2017;155(2):334–47.

[11] Reddy PRK, Kumar DS, Rao ER, Seshiah CV, Sateesh K, Rao KA, et al Environmental sustainability assessment of tropical dairy buffalo farming vis-a-vis sustainable feed replacement strategy. Scientific Reports. 2019;9(1):1–16. 10.1038/s41598-018-37186-2 31728009 PMC6856187

[12] Reddy PRK, Kumar DS, Rao ER, Seshiah CV, Sateesh K, Reddy YPK, et al Assessment of eco-sustainability vis-à-vis zoo-technical attributes of soybean meal (SBM) replacement with varying levels of coated urea in Nellore sheep (Ovis aries). PLOS ONE. 2019;14(8). 10.1371/journal.pone.0220252 PMC6692044 31408459

[13] Flachowsky G, Lebzien P. Possibilities for reduction of nitrogen (N) excretion from ruminants and the need for further research-a review. Landbauforschung Volkenrode. 2006;56(1–2):19–30.

[14] Powell JM. Feed and manure use in low-N-input and high-N-input dairy cattle production systems. Environmental Research Letters. 2014;9(11):115004.

[15] Oenema O. Nitrogen budgets and losses in livestock systems. International Congress Series. 2006;1293:262–71.

[16] Sauvant D, Schmidely P, Daudin J-J, St-Pierre NR. Meta-analyses of experimental data in animal nutrition⋆. Animal. 2008;2(8):1203–14. 10.1017/S1751731108002280 22443733

[17] St-Pierre N. Invited review: Integrating quantitative findings from multiple studies using mixed model methodology. Journal of Dairy Science. 2001;84(4):741–55. 10.3168/jds.S0022-0302(01)74530-4 11352149

[18] Salami SA, Moran CA, Warren HE, Taylor-Pickard J. A Meta-Analysis of the Effects of Slow-Release Urea Supplementation on the Performance of Beef Cattle. Animals. 2020;10(4):657 10.3390/ani10040657 32290182 PMC7223368

[19] Moher D, Liberati A, Tetzlaff J, Altman DG. Preferred reporting items for systematic reviews and meta-analyses: the PRISMA statement. Annals of Internal Medicine. 2009;151(4):264–9. 10.7326/0003-4819-151-4-200908180-00135 19622511

[20] Garcia-Gonzalez R, Tricarico J, Harrison G, Meyer M, McLeod K, Harmon D, et al Optigen® is a sustained release source of non-protein nitrogen in the rumen. Journal of Animal Science. 2007;85:98.

[21] Abdel-Raouf E, Bassiouni M, Ali M, Hassanien H. Effect of Using Slow-Release Urea on Milk Production and its Composition of Lactating Dairy Cows. Journal of Sustainable Agriculture Science. 2017;43:17–26.

[22] Agovino M. Optigen in diets for lactating dairy cows: milk composition and production in an Italian commercial herd. In Scientific poster presented at the 25th Alltech Symposium, Lexington, KY, USA, 17–20 May; Alltech Inc: Nicholasville, KY, USA 2009 10.1016/j.micinf.2009.08.007

[23] Akay V, Tikofsky J, Holtz C, Dawson K. Optigen® 1200: Controlled release of non-protein nitrogen in the rumen. In Proceedings of the 20th Alltech Symposium; Lexington, USA, 23–24 May; Alltech Inc: Nicholasville, KY, USA 2004:pp. 179–85.

[24] Gadegaonkar G, Patil M, Gulvane S, Karambele N, Jagadale S. Effect of Supplementation of Slow Release Non-Protein Nitrogen Compound on the Lactation of Cows. The Indian Journal of Veterinary Sciences and Biotechnology. 2018;14(3):24–7.

[25] Galo E, Emanuele S, Sniffen C, White J, Knapp J. Effects of a polymer-coated urea product on nitrogen metabolism in lactating Holstein dairy cattle. Journal of Dairy Science. 2003;86(6):2154–62. 10.3168/jds.S0022-0302(03)73805-3 12836952

[26] Giallongo F, Hristov AN, Oh J, Frederick T, Weeks H, Werner J, et al Effects of slow-release urea and rumen-protected methionine and histidine on performance of dairy cows. Journal of Dairy Science. 2015;98(5):3292–308. 10.3168/jds.2014-8791 25726096

[27] Inostroza J, Shaver R, Cabrera V, Tricárico J. Effect of diets containing a controlled-release urea product on milk yield, milk composition, and milk component yields in commercial Wisconsin dairy herds and economic implications. The Professional Animal Scientist. 2010;26(2):175–80.

[28] Miranda MS, Arcaro JRP, Netto AS, Silva SdL, Pinheiro MdG, Leme PR. Effects of partial replacement of soybean meal with other protein sources in diets of lactating cows. Animal. 2019;13(7):1403–11. 10.1017/S1751731118002926 30415645

[29] Neal K, Eun J-S, Young A, Mjoun K, Hall J. Feeding protein supplements in alfalfa hay-based lactation diets improves nutrient utilization, lactational performance, and feed efficiency of dairy cows. Journal of Dairy Science. 2014;97(12):7716–28. 10.3168/jds.2014-8033 25262186

[30] Santiago BT, Villela SDJ, Leonel FdP, Zervoudakis JT, Araújo RP, Machado HVN, et al Slow-release urea in diets for lactating crossbred cows. Revista Brasileira de Zootecnia. 2015;44(5):193–9.

[31] Santos J, Pereira M, Dias-Junior G, Bitencourt L, Lopes N, Junior S, et al Response of lactating cows to partial replacement of soybean by optigen or urea. In Scientific poster presented at the 25th Alltech Symposium, Lexington, USA, 17–20 May; Alltech Inc: Nicholasville, KY, USA 2009.

[32] Sinclair L, Blake C, Griffin P, Jones G. The partial replacement of soyabean meal and rapeseed meal with feed grade urea or a slow-release urea and its effect on the performance, metabolism and digestibility in dairy cows. Animal. 2012;6(6):920–7. 10.1017/S1751731111002485 22558962

[33] Souza V, Almeida R, Silva D, Piekarski P, Jesus C, Pereira M. Effects of partial replacement of soybean meal by protected urea on milk yield and composition. Arquivo Brasileiro de Medicina Veterinária e Zootecnia. 2010;62(6):1415–22.

[34] Stewart R, Tricarico J, Harmon D, Chalupa W, McLeod K, Harrison G, et al Influence of Optigen on nitrogen behavior in lactating dairy cows. Journal of Dairy Science. 2008;91(Suppl 1):491.

[35] Tye BM, Yang S-Y, Eun J-S, Young AJ, Hall JO. An investigation of feeding high-moisture corn grain with slow-release urea supplementation on lactational performance, energy partitioning, and ruminal fermentation of dairy cows. Canadian Journal of Animal Science. 2017;97(4):742–52.

[36] Varga G, Ishler V. Effect of optigen on milk production, N balance and diet cost in high producing cows. In Scientific poster presented at the 25th Alltech Symposium, Lexington, USA, 17–20 May; Alltech Inc: Nicholasville, KY, USA 2009.

[37] Vellinga TV, Blonk H, Marinussen M, Van Zeist W, Starmans D. Methodology used in feedprint: a tool quantifying greenhouse gas emissions of feed production and utilization. Wageningen UR Livestock Research, Wageningen, The Netherlands, 2013 1570–8616.

[38] Kebreab E, Strathe A, Dijkstra J, Mills JA, Reynolds CK, Crompton LA, et al, editors. Energy and protein interactions and their effect on nitrogen excretion in dairy cows. Symposium on Energy and Protein Metabolism and Nutrition, Parma, Italy; 2010.

[39] Tomlinson A, Powers W, Van Horn H, Nordstedt R, Wilcox C. Dietary protein effects on nitrogen excretion and manure characteristics of lactating cows. Transactions of the ASAE. 1996;39(4):1441–8.

[40] Weiss WP, Willett L, St-Pierre N, Borger D, McKelvey T, Wyatt D. Varying forage type, metabolizable protein concentration, and carbohydrate source affects manure excretion, manure ammonia, and nitrogen metabolism of dairy cows. Journal of Dairy Science. 2009;92(11):5607–19. 10.3168/jds.2009-2248 19841221

[41] Münnich M, Khiaosa-Ard R, Klevenhusen F, Hilpold A, Khol-Parisini A, Zebeli Q. A meta-analysis of feeding sugar beet pulp in dairy cows: effects on feed intake, ruminal fermentation, performance, and net food production. Animal Feed Science and Technology. 2017;224:78–89.

[42] Wagner J, Davis N. A Meta-Analysis Evaluation of Feeding MGA® to Feedlot Heifers Implanted with TBA. Proceedings, American Society of Animal Science Western Section. 2007;58:3–6.

[43] Begg CB, Mazumdar M. Operating characteristics of a rank correlation test for publication bias. Biometrics. 1994:1088–101. 7786990

[44] Schwab C, Huhtanen P, Hunt C, Hvelplund T. Nitrogen requirements of cattle. Nitrogen and Phosphorus Nutrition of Cattle and Environment: CAB International, Wallingford, UK; 2005 p. 13–70.

[45] Poppy G, Rabiee A, Lean I, Sanchez W, Dorton K, Morley P. A meta-analysis of the effects of feeding yeast culture produced by anaerobic fermentation of Saccharomyces cerevisiae on milk production of lactating dairy cows. Journal of Dairy Science. 2012;95(10):6027–41. 10.3168/jds.2012-5577 22921623

[46] Eugène M, Massé D, Chiquette J, Benchaar C. Meta-analysis on the effects of lipid supplementation on methane production in lactating dairy cows. Canadian Journal of Animal Science. 2008;88(2):331–7.

[47] Huhtanen P, Hristov AN. A meta-analysis of the effects of dietary protein concentration and degradability on milk protein yield and milk N efficiency in dairy cows. Journal of Dairy Science. 2009;92(7):3222–32. 10.3168/jds.2008-1352 19528599

[48] Lawrence D, O’Donovan M, Boland T, Lewis E, Kennedy E. The effect of concentrate feeding amount and feeding strategy on milk production, dry matter intake, and energy partitioning of autumn-calving Holstein-Friesian cows. Journal of Dairy Science. 2015;98(1):338–48. 10.3168/jds.2014-7905 25465538

[49] McNamara S, O’Mara F, Rath M, Murphy J. Effects of different transition diets on dry matter intake, milk production, and milk composition in dairy cows. Journal of Dairy Science. 2003;86(7):2397–408. 10.3168/jds.S0022-0302(03)73834-X 12906058

[50] Connor E. Invited review: Improving feed efficiency in dairy production: challenges and possibilities. Animal. 2015;9(3):395–408. 10.1017/S1751731114002997 25482927

[51] Coleman J, Berry D, Pierce K, Brennan A, Horan B. Dry matter intake and feed efficiency profiles of 3 genotypes of Holstein-Friesian within pasture-based systems of milk production. Journal of Dairy Science. 2010;93(9):4318–31. 10.3168/jds.2009-2686 20723705

[52] Waghorn G, Hegarty R. Lowering ruminant methane emissions through improved feed conversion efficiency. Animal Feed Science and Technology. 2011;166:291–301.

[53] Yan X, Yan B, Ren Q, Dou J, Wang W, Zhang J, et al Effect of slow-release urea on the composition of ruminal bacteria and fungi communities in yak. Animal feed science and technology. 2018;244:18–27.

[54] Li Z, Mu C, Xu Y, Shen J, Zhu W. Changes in the Solid-, Liquid-, and Epithelium-Associated Bacterial Communities in the Rumen of Hu Lambs in Response to Dietary Urea Supplementation. Frontiers in Microbiology. 2020;11:244 10.3389/fmicb.2020.00244 32153533 PMC7046558

[55] Waghorn G, Dewhurst R. Feed efficiency in cattle the contribution of rumen function. Meeting the challenges for pasture-based dairying Victoria: Proceedings of the 3rd Dairy Science Symposium. 2007;111:23.

[56] Foskolos A, Moorby JM. Evaluating lifetime nitrogen use efficiency of dairy cattle: A modelling approach. PLOS ONE. 2018;13(8). 10.1371/journal.pone.0201638 30071098 PMC6072052

[57] Hristov AN, Bannink A, Crompton LA, Huhtanen P, Kreuzer M, McGee M, et al Invited review: Nitrogen in ruminant nutrition: A review of measurement techniques. Journal of Dairy Science. 2019;102(7):5811–52. 10.3168/jds.2018-15829 31030912

[58] Powell J, MacLeod M, Vellinga TV, Opio C, Falcucci A, Tempio G, et al Feed–milk–manure nitrogen relationships in global dairy production systems. Livestock Science. 2013;152(2–3):261–72.

[59] Whelan S, Mulligan F, Pierce K. Nitrogen efficiency in contrasting dairy production systems. Advances in Animal Biosciences. 2013;4(s1):9–14.

[60] Salami S, Luciano G, O’Grady M, Biondi L, Newbold C, Kerry J, et al Sustainability of feeding plant by-products: A review of the implications for ruminant meat production. Animal Feed Science and Technology. 2019;251:37–55.

[61] Hristov AN, Oh J, Firkins J, Dijkstra J, Kebreab E, Waghorn G, et al Special topics—Mitigation of methane and nitrous oxide emissions from animal operations: I. A review of enteric methane mitigation options. Journal of Animal Science. 2013;91(11):5045–69. 10.2527/jas.2013-6583 24045497

[62] Alipour D, Saleem AM, Sanderson H, Brand T, Santos LV, Mahmoudi-Abyane M, et al Effect of combinations of feed-grade urea and slow-release urea in a finishing beef diet on fermentation in an artificial rumen system. Translational Animal Science. 2020;4(2):839–847. 10.1093/tas/txaa013 PMC7201078 32705013

[63] Rebelo LR, Luna IC, Messana JD, Araujo RC, Simioni TA, Granja-Salcedo YT, et al Effect of replacing soybean meal with urea or encapsulated nitrate with or without elemental sulfur on nitrogen digestion and methane emissions in feedlot cattle. Animal Feed Science and Technology. 2019;257:114293.

