# Supplementary figures and images for: Meta-analysis and sustainability of feeding slow-release urea in dairy production
Source: PLoS One. 2021 Feb 12;16(2):e0246922. doi: 10.1371/journal.pone.0246922 (PMC7880434; doi:10.1371/journal.pone.0246922)

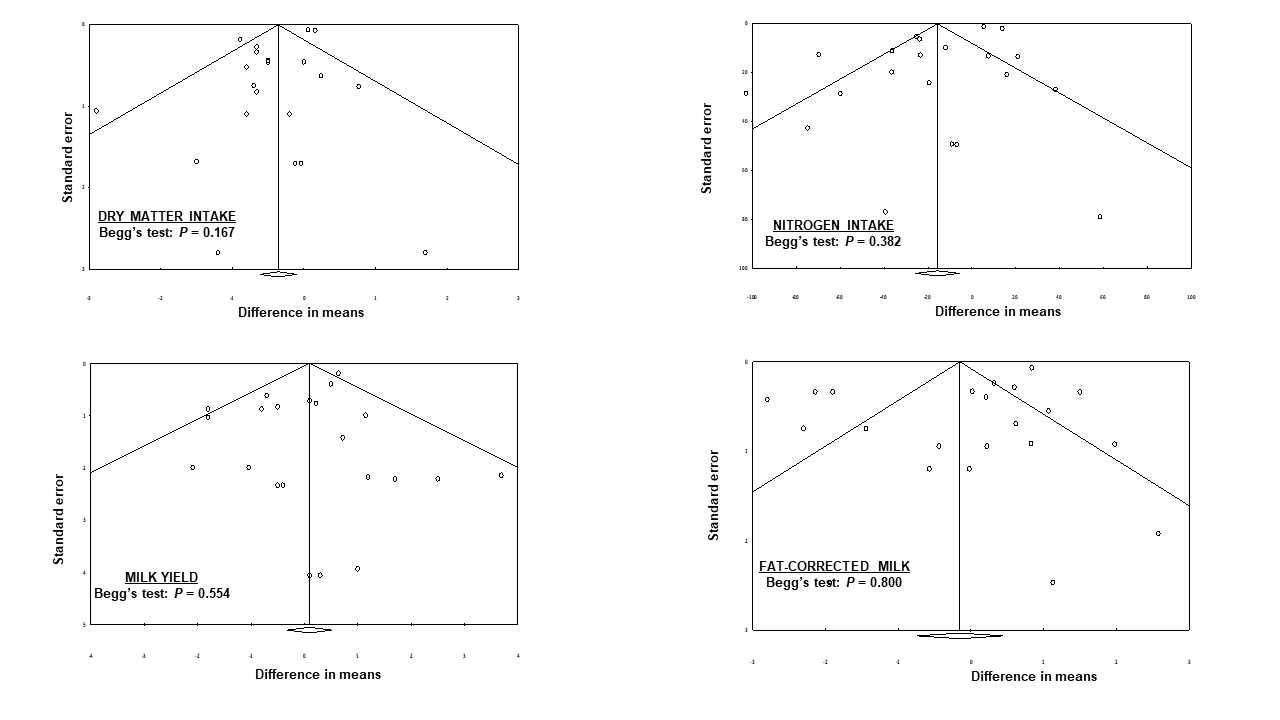

Supplement: S1 Fig — Open circles represent individual study comparisons included in the meta-analysis. (TIF) [file pone.0246922.s001.tif]

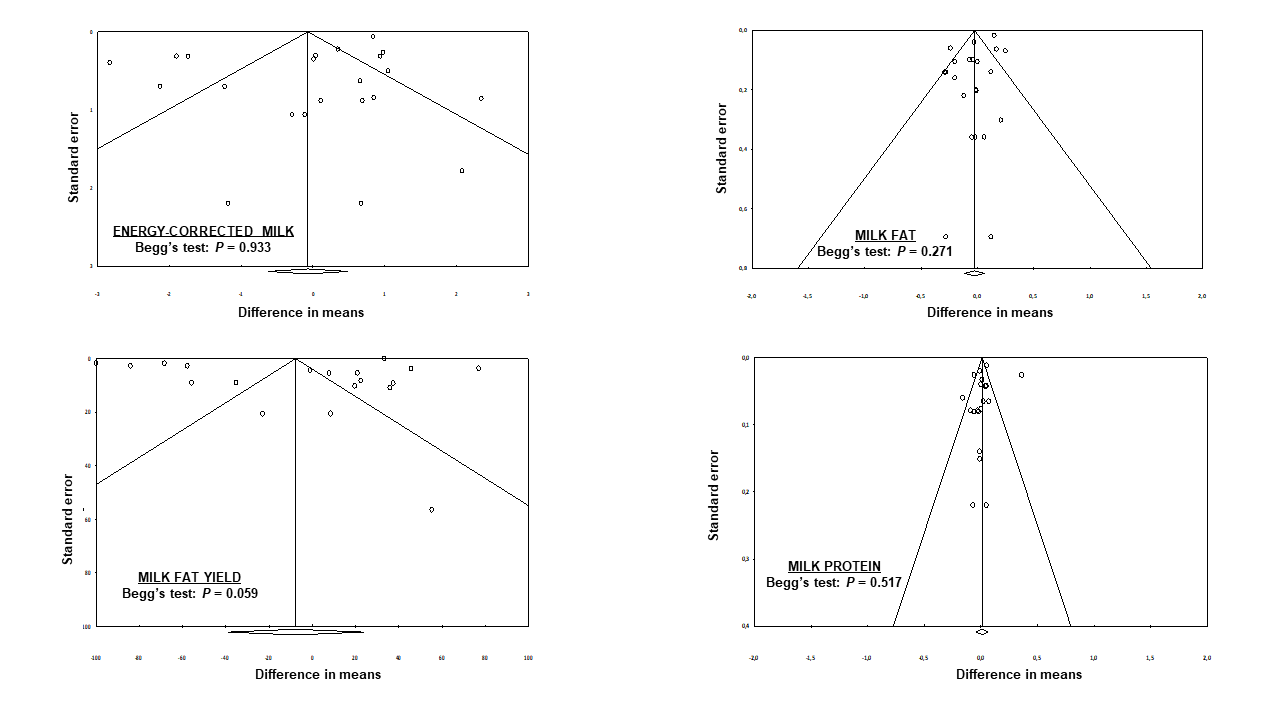

Supplement: S2 Fig — Open circles represent individual study comparisons included in the meta-analysis. (TIF) [file pone.0246922.s002.tif]

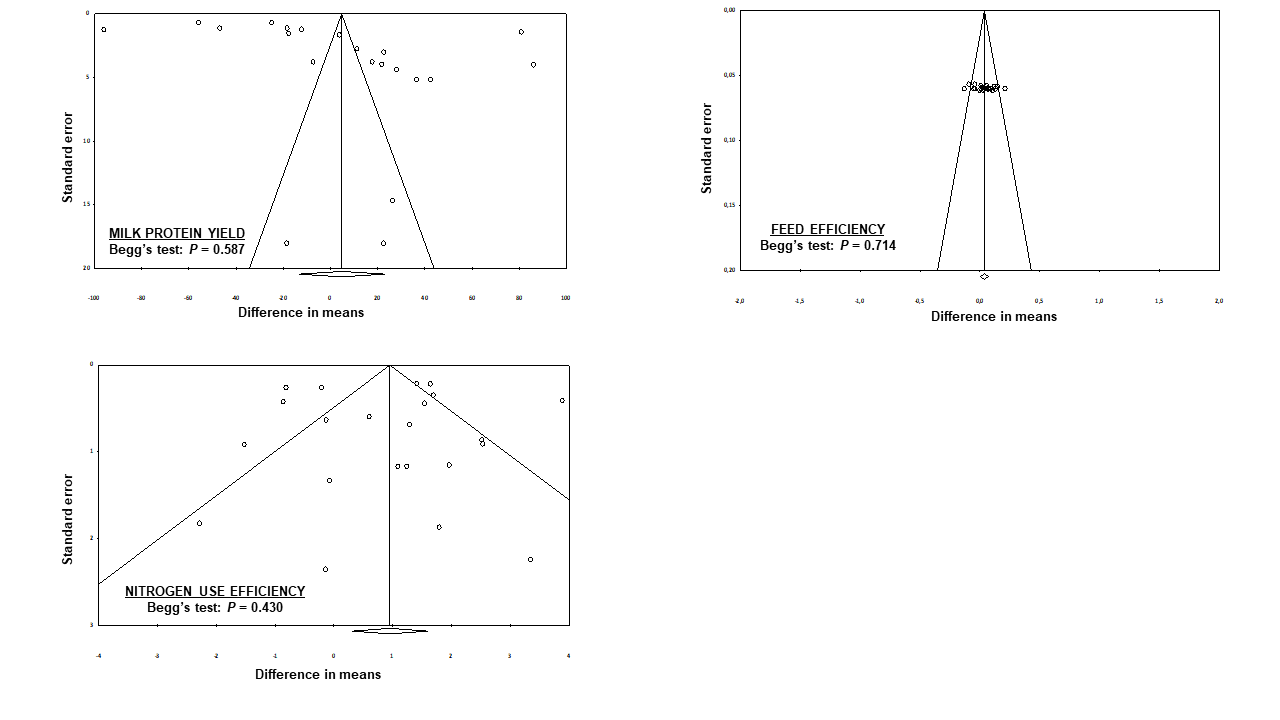

Supplement: S3 Fig — Open circles represent individual study comparisons included in the meta-analysis. (TIF) [file pone.0246922.s003.tif]
