# Supplementary material for: Meta-analysis and sustainability of feeding slow-release urea in dairy production
Source: PLoS One. 2021 Feb 12;16(2):e0246922. doi: 10.1371/journal.pone.0246922 (PMC7880434; doi:10.1371/journal.pone.0246922)
Supplement: S2 Table — (DOCX) [file pone.0246922.s005.docx]

| **S2 Table.** **Carbon footprint (including land use changes) of common feed raw materials used in the studies included in the meta-analysis.** | |
| --- | --- |
| **Item** | **Carbon footprint (g CO_2-_eq/kg)** |
| Soybean meal | 4152 |
| Toasted soybean | 3615 |
| Soybean meal bypass | 4588 |
| Heated soybean seeds | 3615 |
| Rapeseed meal | 1055 |
| Cottonseed | 990 |
| Alfalfa haylage | 217 |
| Alfalfa hay | 217 |
| Alfalfa silage | 106 |
| Linseed meal | 760 |
| Cottonseed cake | 1015 |
| Fish meal | 1283 |
| Corn gluten meal | 991 |
| Maize distillers’ grains | 285 |
| Wheat distillers’ grains | 285 |
| Corn silage | 52 |
| Sorghum silage | 52 |
| Ryegrass hay | 490 |
| Corn meal | 560 |
| High moisture corn | 596 |
| Sugar beet pulp | 357 |
| Corn grain | 596 |
| Corn flake | 600 |
| Ryegrass silage | 292 |
| Wheat straw | 251 |
| Grass haylage | 490 |
| Palm kernel meal | 648 |
| Triticale | 500 |
| Citrus pulp | 701 |
| Wheat bran | 449 |
| Rice bran | 471 |
| Brewers grain | 434 |
| Wheat middlings | 276 |
| Cottonseed hull | 507 |
| Wheat | 455 |
| Soybean hull | 2205 |
| *Optigen | 1136 |
