# Supplementary material for: Meta-analysis and sustainability of feeding slow-release urea in dairy production
Source: PLoS One. 2021 Feb 12;16(2):e0246922. doi: 10.1371/journal.pone.0246922 (PMC7880434; doi:10.1371/journal.pone.0246922)
Supplement: S3 Table — (DOCX) [file pone.0246922.s006.docx]

| **S3 Table. Effect of diet reformulation with slow-release urea (SRU) on the** **average dietary inclusion levels of vegetable protein sources in studies used in the meta-analysis.** | | | | | | |
| --- | --- | --- | --- | --- | --- | --- |
| **Feed ingredient** | **Number of diets** | |  | **Average inclusion level (kg/diet)** | | |
|  | **Control** | **SRU** |  | **Control** | **SRU** | **% Difference** |
| Soybean meal | 16.00 | 18.00 |  | 8.66 | 6.86 | -20.77 |
| Toasted soybean | 2.00 | 1.00 |  | 1.26 | 1.30 | 2.92 |
| SBM bypass | 5.00 | 5.00 |  | 3.02 | 2.36 | -21.86 |
| Heated soybean | 2.00 | 1.00 |  | 9.01 | 9.01 | 0.00 |
| Canola/rapeseed meal | 9.00 | 7.00 |  | 5.42 | 3.84 | -29.16 |
| Cottonseed grain | 6.00 | 6.00 |  | 5.56 | 5.54 | -0.30 |
| Alfalfa haylage | 5.00 | 4.00 |  | 34.11 | 22.97 | -32.66 |
| Alfalfa hay | 5.00 | 7.00 |  | 26.21 | 22.38 | -14.62 |
| Alfalfa silage | 1.00 | 1.00 |  | 93.33 | 91.00 | -2.50 |
| Linseed meal | 1.00 | 2.00 |  | 4.17 | 4.17 | 0.00 |
| Cottonseed cake | 1.00 | 1.00 |  | 10.00 | 3.80 | -62.00 |
| Corn gluten meal | 3.00 | 3.00 |  | 0.64 | 0.37 | -41.95 |
| Corn distillers’ grains | 4.00 | 4.00 |  | 7.42 | 6.03 | -18.73 |
| Wheat distillers’ grains | 1.00 | 1.00 |  | 0.93 | 0.93 | 0.00 |
| All soybean products |  |  |  | 21.95 | 19.53 | -11.03 |
| All alfalfa products |  |  |  | 153.65 | 136.34 | -11.26 |
| CON: control treatment; SRU: slow-release urea treatment | | | | | | |
