# Supplementary material for: Meta-analysis and sustainability of feeding slow-release urea in dairy production
Source: PLoS One. 2021 Feb 12;16(2):e0246922. doi: 10.1371/journal.pone.0246922 (PMC7880434; doi:10.1371/journal.pone.0246922)
Supplement: S4 Table — (DOCX) [file pone.0246922.s007.docx]

| **S4 Table. Effect of diet reformulation with slow-release urea (SRU) on the** **average dietary inclusion levels of vegetable energy and fibre sources in studies used in the meta-analysis.** | | | | | | |
| --- | --- | --- | --- | --- | --- | --- |
| **Feed ingredients** | **Number of diets** | |  | **Average inclusion level (kg/diet)** | | |
|  | **Control** | **Optigen** |  | **Control** | **SRU** | **% Difference** |
| Corn silage | 17.00 | 18.00 |  | 81.30 | 81.15 | -0.18 |
| Sorghum silage | 1.00 | 1.00 |  | 75.00 | 75.00 | 0.00 |
| Ryegrass hay | 8.00 | 7.00 |  | 5.13 | 4.63 | -9.77 |
| Corn meal | 2.00 | 2.00 |  | 6.34 | 7.93 | 25.03 |
| High moisture corn | 2.00 | 2.00 |  | 23.08 | 24.08 | 4.33 |
| Sugar beet pulp | 6.00 | 7.00 |  | 5.42 | 5.79 | 6.75 |
| Corn grain | 12.00 | 13.00 |  | 14.08 | 17.51 | 24.35 |
| Corn flake | 2.00 | 2.00 |  | 19.39 | 19.34 | -0.29 |
| Ryegrass silage | 1.00 | 1.00 |  | 46.41 | 46.41 | 0.00 |
| Wheat straw | 1.00 | 1.00 |  | 10.61 | 10.38 | -2.13 |
| Grass haylage | 3.00 | 4.00 |  | 20.63 | 21.79 | 5.62 |
| Palm kernel meal | 1.00 | 1.00 |  | 0.88 | 0.88 | 0.00 |
| Triticale | 1.00 | 1.00 |  | 17.36 | 17.36 | 0.00 |
| Citrus pulp | 1.00 | 1.00 |  | 13.71 | 17.30 | 26.19 |
| Wheat bran | 3.00 | 4.00 |  | 6.78 | 14.92 | 119.93 |
| Rice bran | 1.00 | 2.00 |  | 6.56 | 6.56 | 0.00 |
| Brewers grain | 1.00 | 1.00 |  | 0.48 | 0.44 | -9.09 |
| Wheat middlings | 0.00 | 3.00 |  | 0.00 | 3.32 | 332.17 |
| Cottonseed hulls | 5.00 | 4.00 |  | 9.03 | 6.51 | -27.93 |
| Wheat | 1.00 | 1.00 |  | 0.96 | 0.96 | 0.00 |
| Soybean hulls | 9.00 | 10.00 |  | 8.82 | 6.99 | -20.79 |
| All corn products |  |  |  | 62.90 | 68.86 | 9.47 |
| All silage products |  |  |  | 202.71 | 202.56 | -0.07 |
| All hay/haylage products |  |  |  | 25.76 | 26.42 | 2.55 |
| CON: control treatment; SRU: slow-release urea treatment | | | | | | |
